# Prognostic evaluation models for primary thyroid lymphoma, based on the SEER database and an external validation cohort

**DOI:** 10.1007/s40618-021-01712-3

**Published:** 2021-12-04

**Authors:** Yunshu Zhu, Sheng Yang, Xiaohui He

**Affiliations:** Department of Medical Oncology, National Cancer Center/National Clinical Research Center for Cancer/Cancer Hospital, Chinese Academy of Medical Sciences and Peking Union Medical College, Beijing, 100021 China

**Keywords:** Incidence, Primary thyroid lymphoma, Prognosis, Nomogram, Competing risk

## Abstract

**Purpose:**

Primary thyroid lymphoma (PTL) is a rare malignancy, and the literature is limited to small case series and case reports. This study aimed to assess the epidemiologic characteristics, survival, and prognostic factors of patients with PTL.

**Methods:**

We analyzed 2215 PTL patients from the Surveillance, Epidemiology, and End Results database medical records, between 1983 and 2015, as the training cohort. We enrolled 105 patients from the Cancer Hospital, Chinese Academy of Medical Sciences, for the external validation cohort. The nomograms for predicting the 1-, 5-, and 10-year overall survival (OS) and lymphoma-specific survival (LSS) were constructed.

**Results:**

PTL incidence steadily increased from 1977 to 1994, with an annual percentage change of 3.2% (95% confidence interval [CI]: 1.2–5.2, *P* < 0.05). The 1-, 5-, and 10-year OS and LSS rates were 84.66%, 71.61%, and 55.95%; and 90.5%, 85.7%, and 82.2%, respectively. Multivariate Cox regression analysis revealed that shorter OS association with age ≥ 60 years (hazard ratio [HR], 3.94; 95% CI 3.31–4.69; *P* < 0.001), unmarried status (HR, 1.55; 95% CI 1.37–1.75; *P* < 0.001), Ann Arbor stage III-IV (HR, 1.55; 95% CI 1.37–1.75; *P* = 0.020), diffuse large B-cell lymphoma (HR, 2.60; 95% CI 1.15–5.87; P = 0.022), and T cell non–Hodgkin lymphoma (HR, 3.53; 95% CI 1.12–11.10; *P* = 0.031). In the multivariate competing-risk analyzes, age, stages III-IV, year of diagnosis, surgery, radiation, chemotherapy, and histology were strongly predictive of PTL-specific risk of death. To estimate the 1-, 5-, and 10-year LSS and OS rates, respectively, nomograms were built. In the validation cohort, the results also confirmed the utility.

**Conclusions:**

This study presents the first prognostic model with an external validation that could help clinicians identify patients with high-risk PTL to improve their prognosis.

**Supplementary Information:**

The online version contains supplementary material available at 10.1007/s40618-021-01712-3.

## Introduction

Primary thyroid lymphoma (PTL) is an uncommon malignancy that arises in the thyroid gland with or without the presence of regional lymph nodes in the neck. This definition excludes lymphomas from other sites of the body that invade the thyroid gland. PTL accounts for 1–5% of all thyroid tumours and 2.5–7% of all extra-nodal lymphomas [1]. PTL is observed more frequently in women aged 50–80 years, with a 2–eightfold rise in prevalence than in men [2]. Fast-growing, painless mass of the neck is the most typical clinical presentation. PTL can also cause obstructive symptoms owing to mass aggregation, such as hoarseness, dyspnea, and dysphagia [3]. About 10% of patients with PTL present with the B symptoms, such as fever, night sweating, and weight loss ≥ 10% within the past 6 months [4]. Additionally, laboratory results have demonstrated that 60–70% of PTL are positive for antithyroid antibodies [5].

PTL is diagnosed either by an ultrasound-guided needle biopsy or by surgical biopsy. Owing to the size of the sample used to conduct immunohistochemistry for its precise subtype validation, more tissue is needed for diagnosing PTL than for diagnosing other thyroid tumors [6–8]. The prevalent histology of PTL is non-Hodgkin’s B-cell lymphoma and includes diffuse large B-cell lymphoma (DLBCL), mucosa-associated lymphoid tissue lymphoma (MALT), and mixed MALT and DLBCL [9]. Previous research have showed that tumor histology and stage were prognostic markers; and a lower stage and extra-nodal marginal zone B-cell lymphoma of MALT were correlated with the best outcomes [1].

Since PTL is a rare disease, there are paucity of randomized controlled treatment-assessment trials and large retrospective population studies available on PTL. Our current understanding of this disease comes primarily from case studies or a small series of retrospective analyzes. Despite argument over effective treatment of PTL, combination of systemic chemotherapy and local radiotherapy is the core approach [9].

Thus, the purpose of this study was to assess the epidemiological characteristics and survival, and to create a prognostic model in patients with PTL.

## Materials and methods

### Data sources

#### Training cohort

The data for PTL patients diagnosed between 1983 and 2015 was extracted using Surveillance, Epidemiology, and End Results (SEER)*STAT software (version 8.3.8) from the SEER database. Site-specific code C73.9 was used to classify lymphoma predominantly confined to the thyroid gland, and histologic codes 9590–9595, 9650–9699, and 9700–9729 were used to identify lymphoma in the International Classification of Diseases for Oncology, 3rd edition (ICD-O3). We excluded from the final analysis patients with no pathologically verified diagnosis, which reported zero-day survival period, unclear survival results, unknown cause of death (COD), unknown race, and unknown stage of Ann Arbor (Supplemental file Fig. 1).

The following factors have been used in the analysis: sex, age, race, year of diagnosis, histologic subtype, laterality, diagnostic confirmation, Ann Arbor stage, marital status, surgery, radiation, chemotherapy, vital status, survival months, cause of death, and cause-specific death classification.

#### External validation cohort

Patients who received PTL treatment at the Cancer Hospital of the Chinese Academy of Medical Sciences between April 1998 and January 2015 were retrospectively evaluated using the same criterion for inclusion and exclusion, and then applied as a validation cohort. Information on follow-up was collected using telephone surveys and archives of results from follow-up visits. This research was approved by the ethics committee of the hospital and owing to its retrospective and non-interventional nature, the need for informed consent was waived. Prior to analysis, medical reports were anonymized and deidentified. The risk variables used were comparable to the training cohort.

### Survival data

The primary endpoints of this study were lymphoma-specific survival (LSS) and overall survival (OS). LSS was identified as the time from the date of diagnosis to the date of death from PTL or the date of the last follow-up. For patients who lived < 1 month yet > 0 days in the SEER database, survival time was reported to be zero months; for these patients, we defined 0.5 months as survival time. OS was defined as the time from the date of diagnosis to the date of death from cause or the date of the last follow-up.

### Statistical analyses

The PTL incidence rates were estimated per 1,000,000 persons and were age-adjusted using SEER ∗ STAT (version 8.3.8) to the 2000 US Standard Population. Using the Join-Point regression analysis program (version 4.8.0.1), annual percentage changes (APCs) were calculated. The Kaplan–Meier approach was used to analyze the OS, and it was also assessed using the log-rank test. In the univariate and multivariate tests, the Cox regression model was applied, and the hazard ratios (HRs) with their 95% confidence intervals (CIs) were reported. To build the nomogram for predicting the 1-, 5-, and 10-year OS, the chosen independent prognostic factors were used. To determine the predictive accuracy and discriminative capabilities of the nomogram, the calibration curve, concordance index (C-index), and time-dependent receiver operating characteristic (ROC) curve were used. Eventually, the findings obtained were validated by comparing them to an outside cohort for validation.

To evaluate the LSS, competing-risk analysis was used. Thus, the event of interest was death attributable to LSS, and death attributable to another cause was the competing risk event. The cumulative incidence function (CIF) was used to evaluate the cumulative incidence of lymphoma-specific mortality, and the discrepancies between the groups were calculated using Grey’s test. In the univariate analysis, predictors with a *P* value of < 0.05 were entered into a multivariate analysis based on the proportional sub-distribution hazard models, and sub-distribution hazard ratios (SHRs) with the corresponding 95% CIs were recorded. Those further validated were chosen to produce the nomogram. Using the C-index and calibration curves, the efficiency of the nomogram was evaluated.

All statistical methods were applied using the R version 4.0.3 software (The R Foundation for Statistical Computing, Vienna, Austria; www.rproject.org). The R package included survival, rms, survminer, cmprsk, rmda, mstate, ggplot2, and pec. All tests were two-sided, and P values of < 0.05 were considered statistically significant.

## Results

### Patient demographics and incidence of PTL

Overall, we identified 2,215 PTL patients as a training cohort from the SEER database between 1983 and 2015. The trend of incidence was observed from 1975 to 2017, with an average APC (AAPC) of 2.5% (95% CI − 2.6 to 7.8, *P* = 0.3). From 1977 to 1994, we found a steadily rising incidence with an APC of 3.2% (95% CI 1.2–5.2, *P* < 0.05) (Supplemental file Fig. 2A). Among the female population, this phenomenon was more apparent (Supplemental file Fig. 2B). The age-adjusted incidence of PTL in 1975 was 0.4/1,000,000 persons and in 1976 it was 0.7/1,000,000 persons. The incidence in 2016 and 2017 was 0.8 and 1.1 per 1,000,000 persons, respectively. Over 49.9% of the patients were diagnosed between 2005 and 2015, and 14.7% were diagnosed between 1983 and 1993. The mean age at diagnosis was 65.62 ± 14.65 (range 5–101) years. The entire cohort consisted of 635 (28.7%) men and 1580 (71.3%) women. Most patients were white (90.2%), married (55.4%), and had Ann Arbor stages I–II disease (87.6%). Patient characteristics are summarized in Table 1. The most prevalent subtypes were DLBCL (58.8%) and MALT (15.5%), followed by follicular lymphoma (FL) (10.2%), non-Hodgkin lymphoma not otherwise specified (NHL-NOS) (6.3%), B cell NHL (4.1%) and malignant lymphoma (3.3%). Only, 1.2% of the PTLs were Hodgkin lymphoma and 0.5% were T-cell NHL. More than half of the patients were administered chemotherapy (62.9%). Overall, 1078 patients with PTL (48.7%) underwent radiation, and 970 (43.8%) underwent surgery. In the validation cohort, 60% of the patients had DLBCL, and 86.7% had Ann Arbor stages I–II disease, most patients underwent surgery (75.2%), and most received chemotherapy (93.3%). The features of PTL patients in the validation cohort are summarized in Table 2.

**Table 1:** Characteristics of primary thyroid lymphoma patient who diagnosed in SEER 18 registries, 1983–2015

Characteristic	No. of patients	Percentage (%)
Total	2215	100
Age at diagnosis, years		
Mean ± SD	65.62 ± 14.65	–
Median (range)	67 (5–101)	–
Gender		
Female	1580	71.3
Male	635	28.7
Race		
White	1999	90.2
Black	42	1.9
Others	174	7.9
Years of diagnosis		
1983–1993	325	14.7
1994–2004	785	35.4
2005–2015	1105	49.9
Marital status		
Married	1227	55.4
Unmarried	892	40.3
Unknown	96	4.3
Histology		
HL	27	1.2
DLBCL	1303	58.8
FL	226	10.2
B cell NHL^a^	90	4.1
T cell NHL^b^	12	0.5
ML	74	3.3
NHL, NOS	139	6.3
MALT	344	15.5
Laterality		
Bilateral	6	0.3
Only one side	2209	99.7
Ann Arbor stage		
Stage I–II	1941	87.6
Stage III–IV	274	12.4
Surgery		
No/unknown	1245	56.2
Perform	970	43.8
Radiation		
No/unknown/refused	1137	51.3
Perform	1078	48.7
Chemotherapy		
No/unknown	821	37.1
Perform	1394	62.9

*HL* Hodgkin lymphoma, *FL* follicular lymphoma, *NHL* non–Hodgkin lymphoma, *NOS* not otherwise specified, *MALT* mucosal-associated lymphoid tissue, *DLBCL* diffuse large B-cell lymphoma, *FL* follicular lymphomas, *ML* Malignant lymphoma

^a^Included Burkitt's lymphoma, mantle cell lymphoma, chronic lymphocytic leukemia/small lymphocytic lymphoma, lymphoplasmacytic lymphoma

^b^Included peripheral T-cell lymphoma, anaplastic large cell lymphoma (ALK-positive)

**Table 2:** Patients’ demographics and clinical characteristics of validation cohort of patients with PTL

Characteristic	No. of patients	Percentage (%)
Total	105	100
Age at diagnosis, years		
Mean ± SD	59.1 ± 14.70	–
Median (range)	62 (10–83)	–
Gender		
Female	73	69.5
Male	32	30.5
Marital status		
Married	86	81.9
Unmarried	19	18.1
Histology		
HL	3	2.9
DLBCL	63	60
FL	4	3.8
B cell NHL^a^	6	5.7
T cell NHL^b^	1	1.0
MALT	28	26.7
Ann Arbor stage		
Stage I–II	91	86.7
Stage III–IV	14	13.3
Surgery		
No	26	24.8
Perform	79	75.2
Radiation		
No	45	42.9
Perform	60	57.1
Chemotherapy		
No	7	6.7
Perform	98	93.3

^a^Included Burkitt's lymphoma, mantle cell lymphoma, chronic lymphocytic leukemia/small lymphocytic lymphoma

^b^Included peripheral T-cell lymphoma, anaplastic large cell lymphoma (ALK-positive)

### Survival analysis

Supplemental file Fig. 3A, B demonstrates the OS and LSS of PTL patients. There were 1,128 (50.9%) censored events and 1,087 (49.1%) deaths. The 1-, 5-, and 10-year OS rates were 84.66%, 71.61%, and 55.95%, respectively. The Kaplan–Meier estimate of median OS was 143 months (95% CI 136–153). The best 5-year OS rates were observed among patients with HL (88.9%), MALT (84.7%), and FL (83.2%). The 5-year OS rates in patients with B-cell NHL, DLBCL, and T-cell NHL were 72.5%, 67.1%, and 65.6%, respectively, which were similar to those in patients with NHL-NOS (63.7%) and malignant lymphoma (63.24%). Furthermore, the OS Kaplan–Meier curves for the major PTL subtypes are presented in Supplemental file Fig. 4.

Patients stratified by years of diagnosis, age, surgery, Ann Arbor stage, radiation, chemotherapy, and marital status also had the Kaplan–Meier survival studies conducted. Compared to patients diagnosed between 1983 and 1993, OS was significantly improved in patients diagnosed between 2005 and 2015 (Supplemental file Fig. 4). We observed that age ≥ 60 years, unmarried status, and Ann Arbor stages III-IV disease were significantly associated with inferior OS (Supplemental file Fig. 4). For treatment options, OS was significantly higher in patients who received chemotherapy or surgery or radiation than in those who did not. (Supplemental file Fig. 4).

In the univariate analysis, age, year of diagnosis, histological subtype, Ann Arbor stage, marital status, surgery, chemotherapy, and radiation were significantly associated with OS. The multivariate analysis then incorporated these significant factors that were extracted from the univariate Cox regression analysis. The multivariate Cox regression analysis indicated that independent predictors of OS were marital status, age, Ann Arbor stage, histological subtype, surgery, chemotherapy, and radiation (Table 3). Shorter OS was associated with unmarried status (HR, 1.55; 95% CI 1.37–1.75; *P* < 0.001), age ≥ 60 years (HR, 3.94; 95% CI 3.31–4.69; *P* < 0.001), Ann Arbor stage III-IV (HR, 1.55; 95% CI 1.37–1.75; *P* = 0.020), the pathological subtype of DLBCL (HR, 2.60; 95% CI 1.15–5.87; *P* = 0.022) and T-cell NHL (HR, 3.53; 95% CI 1.12–11.10; *P* = 0.031), while undergoing surgery (HR, 0.77; 95% CI 0.66–0.89; *P* < 0.001), receiving radiation (HR, 0.80; 95% CI 0.70–0.90; *P* < 0.001), and receiving chemotherapy (HR, 0.69; 95% CI 0.60–0.79; *P* < 0.001) were correlated with better OS.

**Table 3:** Multivariable Cox regression model for OS and multivariable Fine & Gray regression model for LSS among PTL patients who diagnosed in SEER 18 registries, 1983–2015

Variable	OS			LSS		
	HR	95% CI	*P*	SHR	95% CI	*P*
Age, years (≤ 60 vs. > 60)	3.94	3.31–4.69	< 0.001	2.52	1.89–3.37	< 0.001
Year of diagnosis						
1983–1993	–				Ref.	
1994–2004	–			0.89	0.68–1.17	0.39
2005–2015	–			0.64	0.47–0.87	0.005
Histology						
HL	Ref.					
DLBCL	2.60	1.15–5.87	0.022	1.02	0.37–2.80	0.97
FL	1.48	0.64–3.42	0.358	0.65	0.23–1.88	0.43
B cell NHL	2.06	0.86–4.92	0.103	0.55	0.17–1.78	0.32
T cell NHL	3.53	1.12–11.10	0.031	0.91	0.15–5.66	0.92
ML	2.74	1.17–6.43	0.020	1.03	0.35–3.04	0.95
NHL, NOS	2.85	1.23–6.61	0.015	1.15	0.39–3.36	0.8
MALT	1.41	0.60–3.26	0.424	0.25	0.08–0.77	0.016
Marital status						
Married	Ref.					
Unmarried	1.55	1.37–1.75	< 0.001	1.12	0.91–1.38	0.29
Unknown	0.95	0.68–1.34	0.787	0.30	0.12–0.76	0.01
Ann Arbor stage						
Stage I–II	Ref.					
Stage III–IV	1.23	1.03–1.47	0.020	1.61	1.23–2.12	< 0.001
Surgery						
No/unknown	Ref.					
Perform	0.77	0.66–0.89	< 0.001	0.76	0.59–0.98	0.031
Radiation						
No/unknown/refused	Ref.					
Perform	0.80	0.70–0.90	< 0.001	0.78	0.63–0.97	0.023
Chemotherapy						
No/unknown	Ref.					
Perform	0.69	0.60–0.79	< 0.001	0.67	0.53–0.85	< 0.001

### PTL-specific survival

Overall, 373 (16.8%) patients died of PTL-related causes, while 714 (32.2%) died of other causes, and the remaining 1128 (50.9%) patients are alive. The 1-, 5-, and 10-year LSS rates were 90.5%, 85.7%, and 82.2%, respectively. Considering other-than-PTL deaths as a competing risk, the univariate analyses of lymphoma-specific mortality were carried out. Age, marital status, year of diagnosis, histological subtype, Ann Arbor stage, and surgery were all associated with LSS (Supplemental file Fig. 5, *P* < 0.001). The multivariate analysis was conducted using the CIF of patients with risk factors for PTL-specific death (*P* ≤ 0.05). Patients with the following features have a higher PTL-specific risk of death in multivariate competing-risk analyses: age ≥ 60 years (SHR, 2.52; 95% CI 1.89–3.37; *P* < 0.001), and Ann Arbor stage III-IV (SHR, 1.61; 95% CI 1.23–2.12; *P* < 0.001). While, diagnosed in 2005–2015 (SHR, 0.64; 95% CI 0.47–0.87; *P* = 0.005), undergoing surgery (SHR, 0.76; 95% CI 0.59–0.98; *P* = 0.031), receiving radiation (SHR, 0.78; 95% CI 0.63–0.97; *P* = 0.023), receiving chemotherapy (SHR, 0.67; 95% CI 0.53–0.85; *P* < 0.001) and having MALT as a pathological subtype (SHR, 0.25; 95% CI 0.08–0.77; *P* = 0.016) were associated with a better LSS (Table 3).

### Construction and validation of the nomograms

**Fig. 1:**
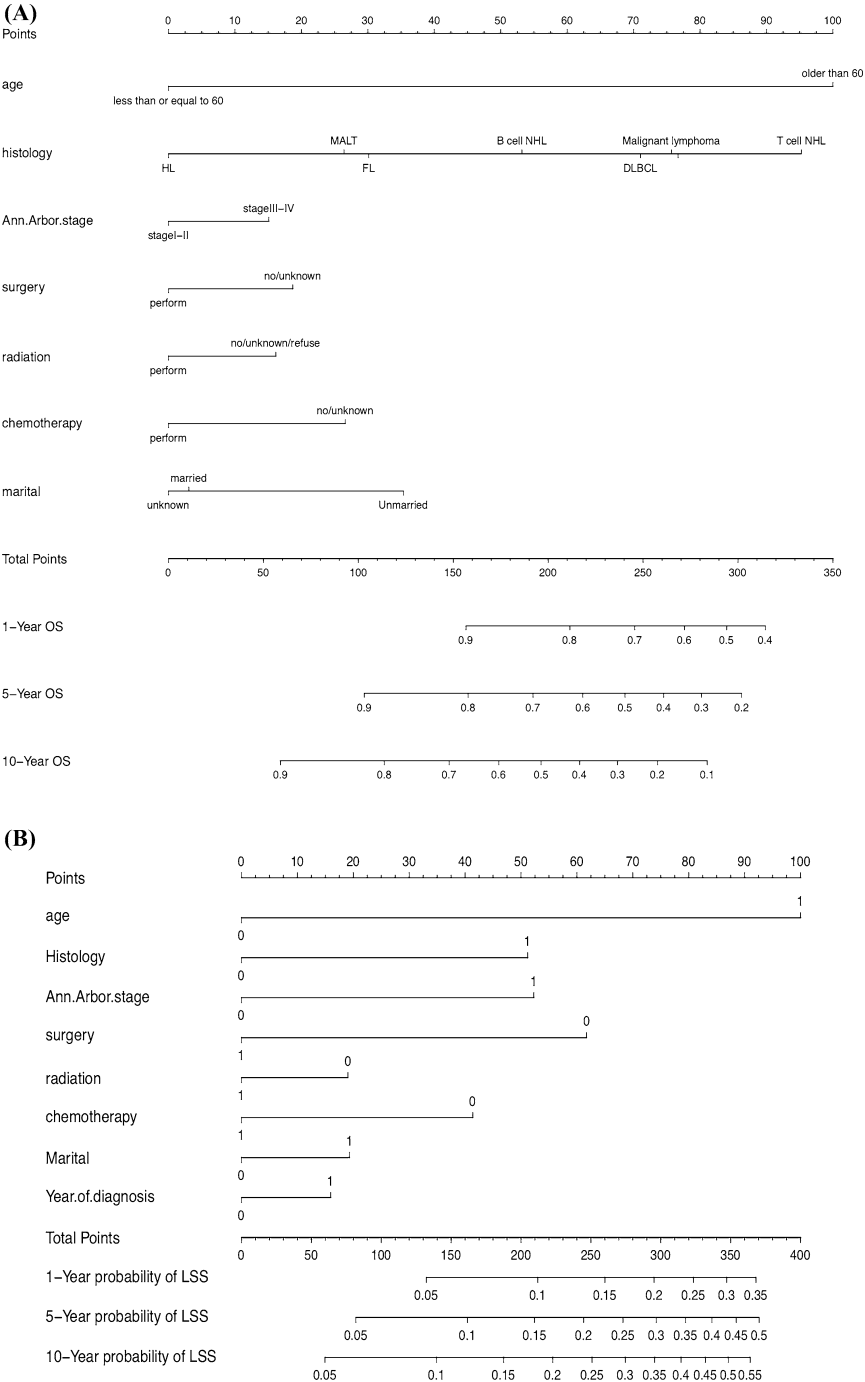
Prognostic nomograms of 1-, 5-, and 10-year **A** OS and **B** LSS

**Fig. 2:**
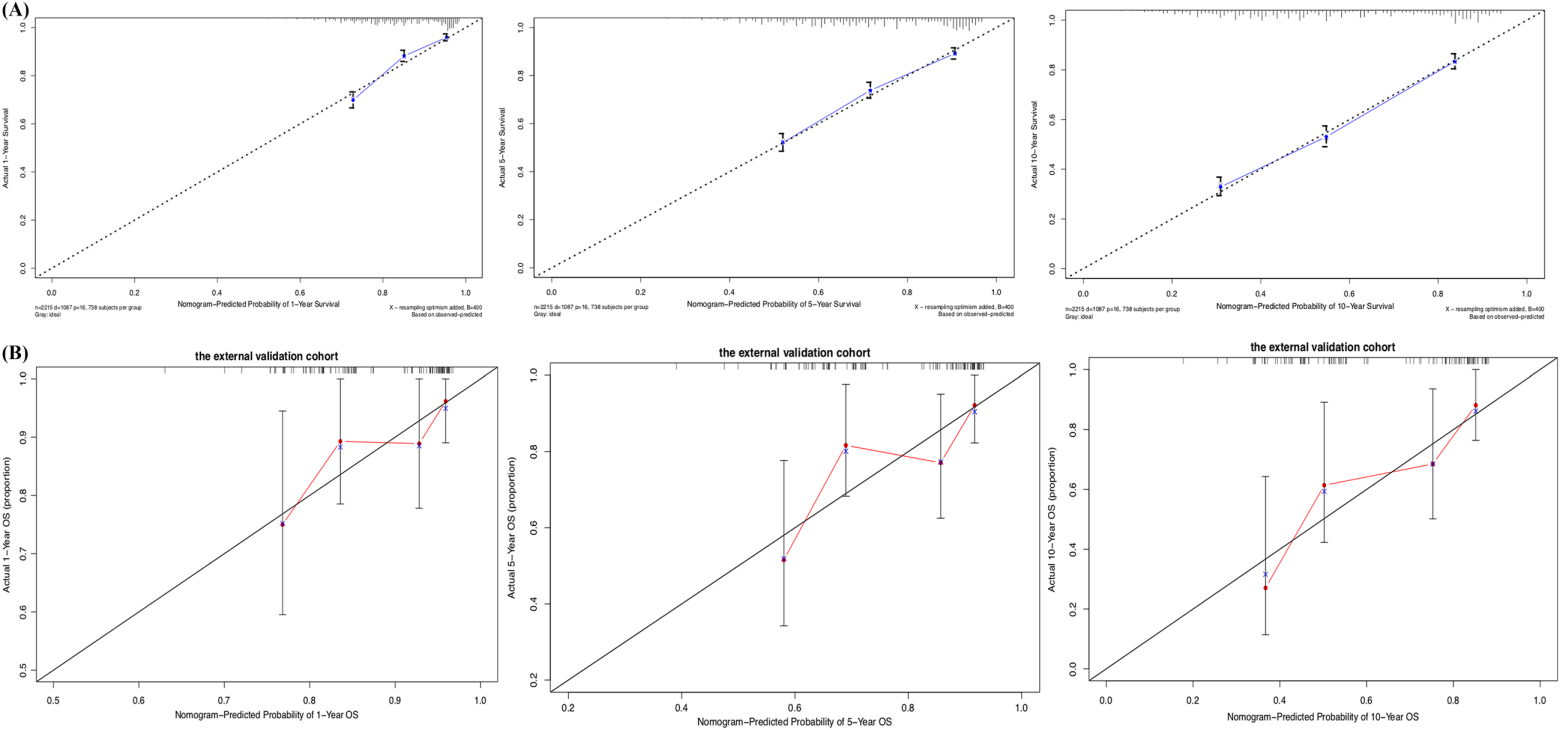
Calibration curves predicting the 1-, 5-, and 10-year OS of patients in the training cohort (**A**) and the external validation cohort (**B**)

**Fig. 3:**
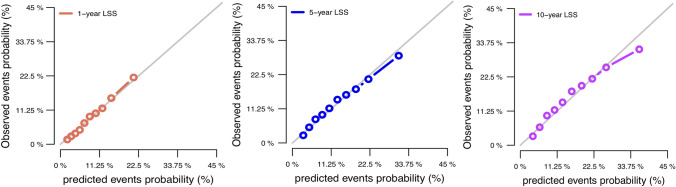
Calibration plots of the nomogram prediction of 1-, 5-, and 10-year LSS of patients with PTL

**Fig. 4:**
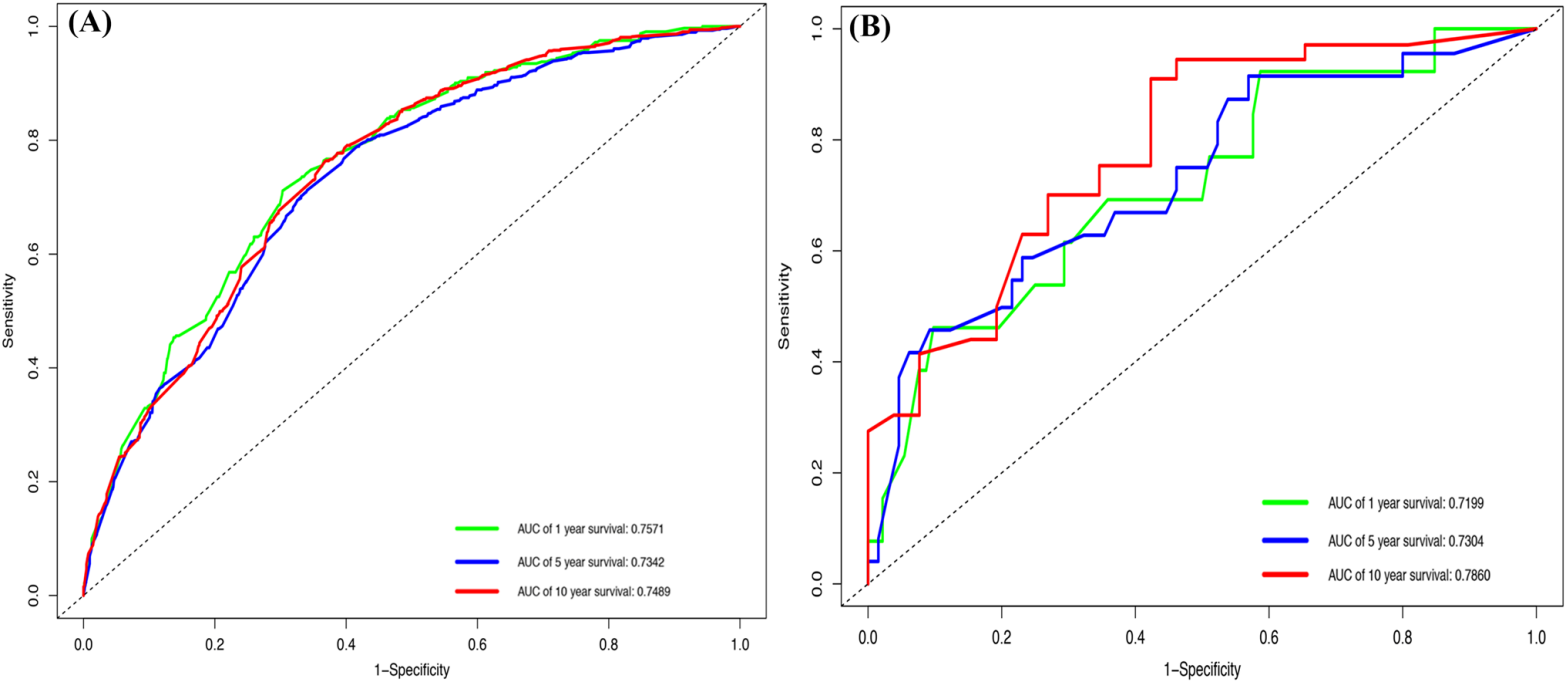
ROC curves and AUCs at 1, 5, and 10-year OS in the training cohort (**A**) and the external validation cohort (**B**) were used to estimate the prognostic accuracy of the nomogram

All the validated predictors were integrated to develop prognostic nomograms based on OS and PTL-specific mortality. Figure 1A, B presents the OS and LSS nomogram at 1-, 5-, and 10-year, respectively. Each model showed relatively good discriminative ability, with a C-index of 0.714 (95% CI 0.696–0.732) for the OS nomogram and 0.690 (95% CI 0.687–0.693) for the LSS nomogram. Analogously, the calibration curves demonstrated outstanding consistency between the nomogram estimate and actual OS (Fig. 2A) or LSS (Fig. 3). As evaluated using ROC curve (Fig. 4A), the OS nomogram presented an area under the curve (AUC) value of 0.757, 0.734, and 0.749 for 1-, 5-, and 10-year OS, respectively. This indicates that there was a high sensitivity and specificity in the OS nomogram.

In the external cohort, the C-index value for the OS predictor nomogram was 0.705 (95% CI 0.611–0.800), suggesting strong discriminative ability. The calibration curves also demonstrated outstanding accuracy between the nomogram prediction and actual OS (Fig. 2B). The ROC curve (Fig. 4B) provided an AUC values of 0.720, 0.730, and 0.786 for 1-, 5-, and 10-year OS, respectively.

## Discussion

PTL is a rare malignant entity that the prognostic factors have not been thoroughly established. There is presently no research presented on the prevalence, characteristics, survival, and prognosis of PTL. Our study established the first prognostic model for predicting OS and LSS, and this was the first PTL study to use data from the SEER database to the best of our knowledge between 1983 and 2015. The nomograms can help doctors to identify high-risk patients by combining clinical, pathological, and biological variables, and then provide an individualized risk estimation and treatment for each patient. Such findings may represent the real-world treatment of patients with PTL. Our study reported the 1-, 5-, and 10-year OS rates of 84.66%, 71.61%, and 55.95%, respectively, and corresponding LSS rates of 90.5%, 85.7%, and 82.2%, respectively, consistent with previous study reports. Victoria Vardell Noble et al. used the National Cancer Database (NCDB) and reported a 75% 5-year OS [10], which ranged from 66 to 74% in other studies [11, 12]. Therefore, the nomograms based on multiple independent risk factors established in this study may be of practical clinical significance in improving the survival and prognosis of PTL patients.

In our study, the incidence trend was observed with an AAPC of 2.5% (95% CI − 2.6 to 7.8, *P* = 0.3) from 1975 to 2017. Of note, we found an upward trend from 1977 to 1994 in the incidence of PTL. Increasing trends in OS and LSS were reported over the past 37 years in more than 85.3% of patients with PTL diagnosed after 1993, which may have benefited from progress in diagnosis modalities and systematic treatment methods. We identified that 66.4% of PTL patients were > 60 years old, which is similar to the prior NCDB reports [10]. Most patients reported in the literature were women, with some studies reporting a female-to-male ratio of 3:1 [11]. We affirm that female predominance appears to exist as the ratio of female to male cases was 2.5:1 in our study, indicating the potential mechanism of sex hormones in PTL pathogenesis. Most PTLs described in the literature were associated with NHL derived from B-cells, including DLBCL or MALT [1, 9, 10, 13]. This result accorded with our finding in which DLBCL (58.8%) was the most common histological subtype, followed by MALT (15.5%) and FL (10.2%). Moreover, we report that the proportion of HL and T-cell NHL was 1.2% and 0.5%, respectively; these subtypes were rarely observed in other studies. A study in 2015, reported that 92.1% of patients had Ann Arbor stages I-II disease [9], this result is similar to our finding that 87.6% were Ann Arbor stages I-II. In 80% of cases, PTL is manifested by a fast-growing thyroid nodule [14], which may partly explain why the majority of PTL cases could be diagnosed at an early stage.

The treatment of PTL is determined using several factors, such as age, stage, histological subtypes and symptoms. The combination of systemic chemotherapy and local–regional radiotherapy is the foundation of treatment. The conventional treatment for NHL involves an anthracycline-based CHOP regimen and rituximab. Single chemotherapy or radiotherapy alone has been administered for indolent lymphomas, while combination therapy is recommended for aggressive lymphomas [12]. Based on the available research, the combined modality treatment achieved a better outcome for OS and disease-free survival than single modality therapy. The function of surgery, however, is not adequately determined. A research performed by the Mayo Clinic showed that patients undergoing diagnostic biopsy plus adjuvant chemotherapy alone had a more effective complete remission than those undergoing debulking plus adjuvant therapy [15]. Our evidence validated the correlation between surgery or radiation or chemotherapy and better survival of patients with PTL. Therefore, the best management for PTL still warrants further study, as most analysis were based on limited sample sizes, and a large, randomized controlled trial is still needed.

In our study, we identified seven independent risk factors for OS, namely, age, histology, Ann Arbor stage, surgery, chemotherapy, radiation, and marital status, using the univariate and multivariate Cox analyses. The independent prognostic factors in our study were consistent with those reported in previous studies. Chai et al. found that patients with early stage, and MALT had better survival outcomes [9]. DiBiase et al. demonstrated that a shorter OS was related to the higher age [16]. Graff-Baker et al. demonstrated that lack of radiation or surgery was independently linked to lower disease-specific survival. The NCDB database study by Vardell Noble et al. supports these findings, where significant improvement was achieved with multiagent and single-agent chemotherapy than without chemotherapy [10]. A nomogram of 1-, 5-, and 10-year OS was established based on the above independent risk factors. Internal and external validations also supported the use of the prognostic evaluation model. In addition, to make our findings more applicable in clinical practice, we built a quantitative competitive risk nomogram to estimate their 1-, 5-, and 10-year LSS. In our model, age, Ann Arbor stage, year of diagnosis, histology, surgery, radiation, chemotherapy, and marital status were strong predictors of LSS. To the best of our knowledge, this work is the first competing risk quantitative nomogram established to predict the LSS of PTL with good prognostic efficiency. The competitive risk estimates generated by the nomogram can instruct clinical decision-making, especially when evaluating less active treatment.

There are some limitations to our analysis. First, this study was restricted by its retrospective nature, which may have led to an unavoidable bias. Prospective research with a large-scale sample size would not be practicable, given the rarity of PTL. Although this analysis used the data in the SEER software, the results were also validated using a real-world cohort, and the possible retrospective shortcomings were entirely accounted for by a rigorous statistical evaluation. Second, data on specific chemotherapy regimens and details on remission, and relapse were not documented in the SEER database. Chemotherapy administration described in the SEER data is stated as either yes or no/unknown. Nonetheless, the efficacy of multiple regimens is beyond the scope of this thesis. Given all this, all studies using the SEER dataset have these shortcomings in common. Despite these drawbacks, the SEER dataset remains a powerful source for researching such an unusual disease. Important insights into PTL, and valuable knowledge on incidence, survival outcome, and prognosis were still generated by our research.

To summarize, PTL is an unusual lymphoma that is reported most often in elderly women. The first precise and realistic OS and LSS nomograms were created and validated, which could help physicians reliably predict the prognosis and build optimum management.

## Supplementary Information

Below is the link to the electronic supplementary material.

Supplemental file figure 1 | A flowchart of patient selection for the current study

Supplemental file figure 2 | (A) Annual age-adjusted incidence of primary thyroid lymphoma patients from 1975 to 2017. (B) Annual age-adjusted incidence of male and female primary thyroid lymphoma patients from 1975 to 2017

Supplemental file figure 3 | Survival analysis of primary thyroid lymphoma: (A) OS and (B) LSS were shown for all patients

Supplemental file figure 4 | Kaplan–Meier survival analysis of overall survival according to (A) age, (B) year of diagnosis, (C) surgery, (D) radiation, (E) chemotherapy, (F) marital, (G) histology, (H) stage

Supplemental file figure 5 | Comparison of the influence of different variables on lymphoma-specific death and non-lymphoma-specific death using Gray’s test. (A) age, (B) year of diagnosis, (C) surgery, (D) radiation, (E) chemotherapy, (F) marital, (G) histology, (H) stage

Supplementary file6 (DOCX 14 kb)

## Data Availability

Publicly available datasets were analyzed in this study. This data can be found in the SEER database (https://seer.cancer.gov/).

## References

[1] Derringer GA (2000). Malignant lymphoma of the thyroid gland: a clinicopathologic study of 108 cases. Am J Surg Pathol.

[2] Demir H (2018). Primary diffuse large b cell lymphoma of thyroid gland; germinal center and non-germinal center B types: experience of a single center. Indian J Surg.

[3] Bostanci H (2017). Eleven patients with primary thyroid lymphoma: a single center experience. Turk J Med Sci.

[4] Green LD, Mack L, Pasieka JL (2006). Anaplastic thyroid cancer and primary thyroid lymphoma: a review of these rare thyroid malignancies. J Surg Oncol.

[5] Niitsu N (2007). Prognostic impact of chromosomal alteration of 3q27 on nodal B-cell lymphoma: correlation with histology, immunophenotype, karyotype, and clinical outcome in 329 consecutive patients. Leuk Res.

[6] Alzouebi M (2012). Primary thyroid lymphoma: the 40 year experience of a UK lymphoma treatment centre. Int J Oncol.

[7] Matsuda M (1987). Fine-needle aspiration cytology of malignant lymphoma of the thyroid. Diagn Cytopathol.

[8] Sharma A (2016). Clinical presentation and diagnostic challenges of thyroid lymphoma: a cohort study. Thyroid.

[9] Chai YJ (2019). Clinicopathological characteristics and treatment outcomes of 38 cases of primary thyroid lymphoma: a multicenter study. Ann Surg Treat Res.

[10] Vardell Noble V (2019). Primary thyroid lymphoma: an analysis of the national cancer database. Cureus.

[11] Graff-Baker A (2009). Prognosis of primary thyroid lymphoma: demographic, clinical, and pathologic predictors of survival in 1,408 cases. Surgery.

[12] Onal C (2011). Treatment results and prognostic factors in primary thyroid lymphoma patients: a rare cancer network study. Ann Oncol.

[13] Cha H (2013). Patterns of care and treatment outcomes for primary thyroid lymphoma: a single institution study. Radiat Oncol J.

[14] Pavlidis ET, Pavlidis TE (2019). A review of primary thyroid lymphoma: molecular factors, diagnosis and management. J Invest Surg.

[15] Pyke CM (1992). Non-Hodgkin's lymphoma of the thyroid: is more than biopsy necessary?. World J Surg.

[16] DiBiase SJ (2004). Outcome analysis for stage IE and IIE thyroid lymphoma. Am J Clin Oncol.

