# Supplementary figures and images for: Prognostic evaluation models for primary thyroid lymphoma, based on the SEER database and an external validation cohort
Source: J Endocrinol Invest. 2021 Dec 4;45(4):815–24. doi: 10.1007/s40618-021-01712-3 (PMC8918170; doi:10.1007/s40618-021-01712-3)

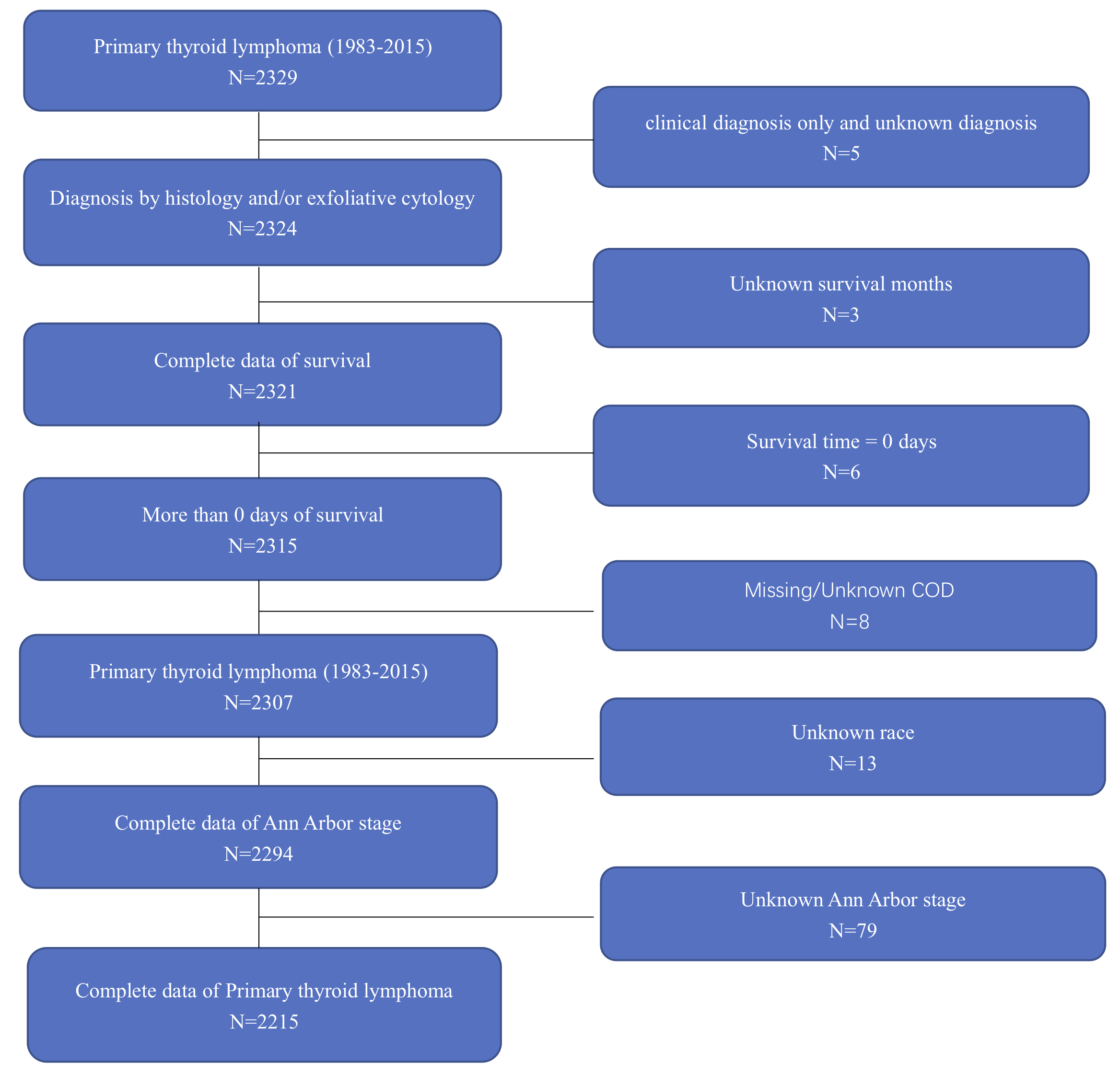

Supplement: Supplementary file 1 — Supplemental file figure 1 | A flowchart of patient selection for the current study [file 40618_2021_1712_MOESM1_ESM.tif]

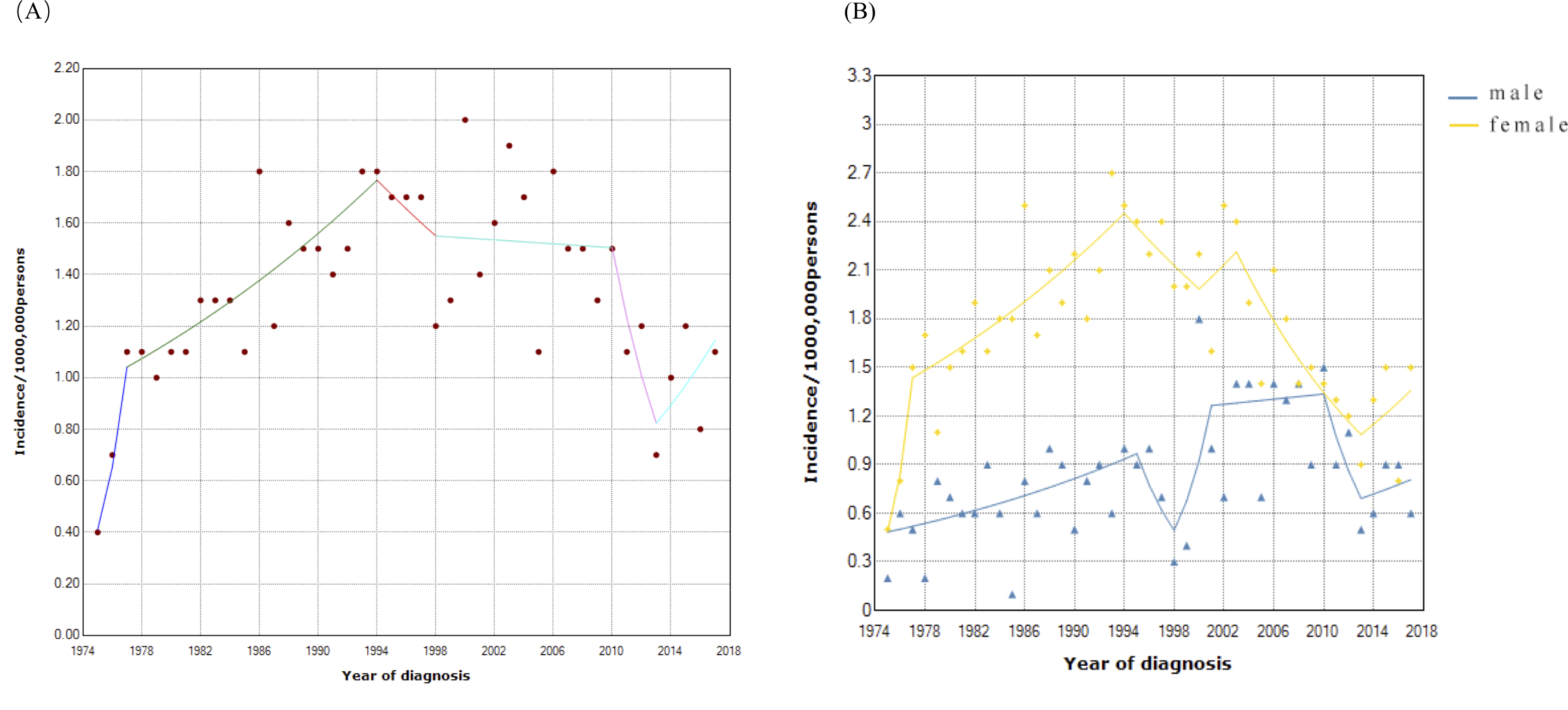

Supplement: Supplementary file 2 — Supplemental file figure 2 | (A) Annual age-adjusted incidence of primary thyroid lymphoma patients from 1975 to 2017. (B) Annual age-adjusted incidence of male and female primary thyroid lymphoma patients from 1975 to 2017 [file 40618_2021_1712_MOESM2_ESM.tif]

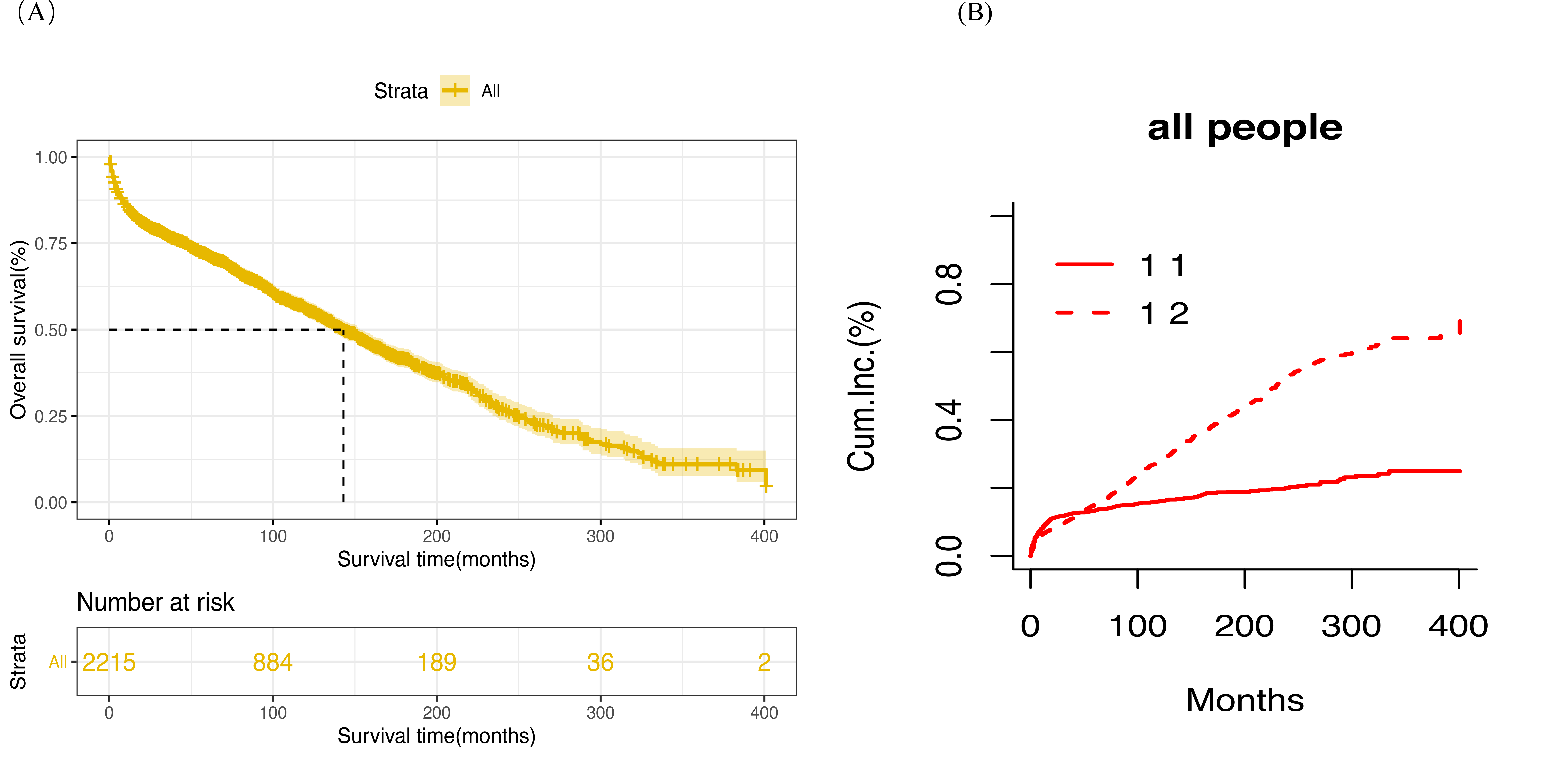

Supplement: Supplementary file 3 — Supplemental file figure 3 | Survival analysis of primary thyroid lymphoma: (A) OS and (B) LSS were shown for all patients [file 40618_2021_1712_MOESM3_ESM.tif]

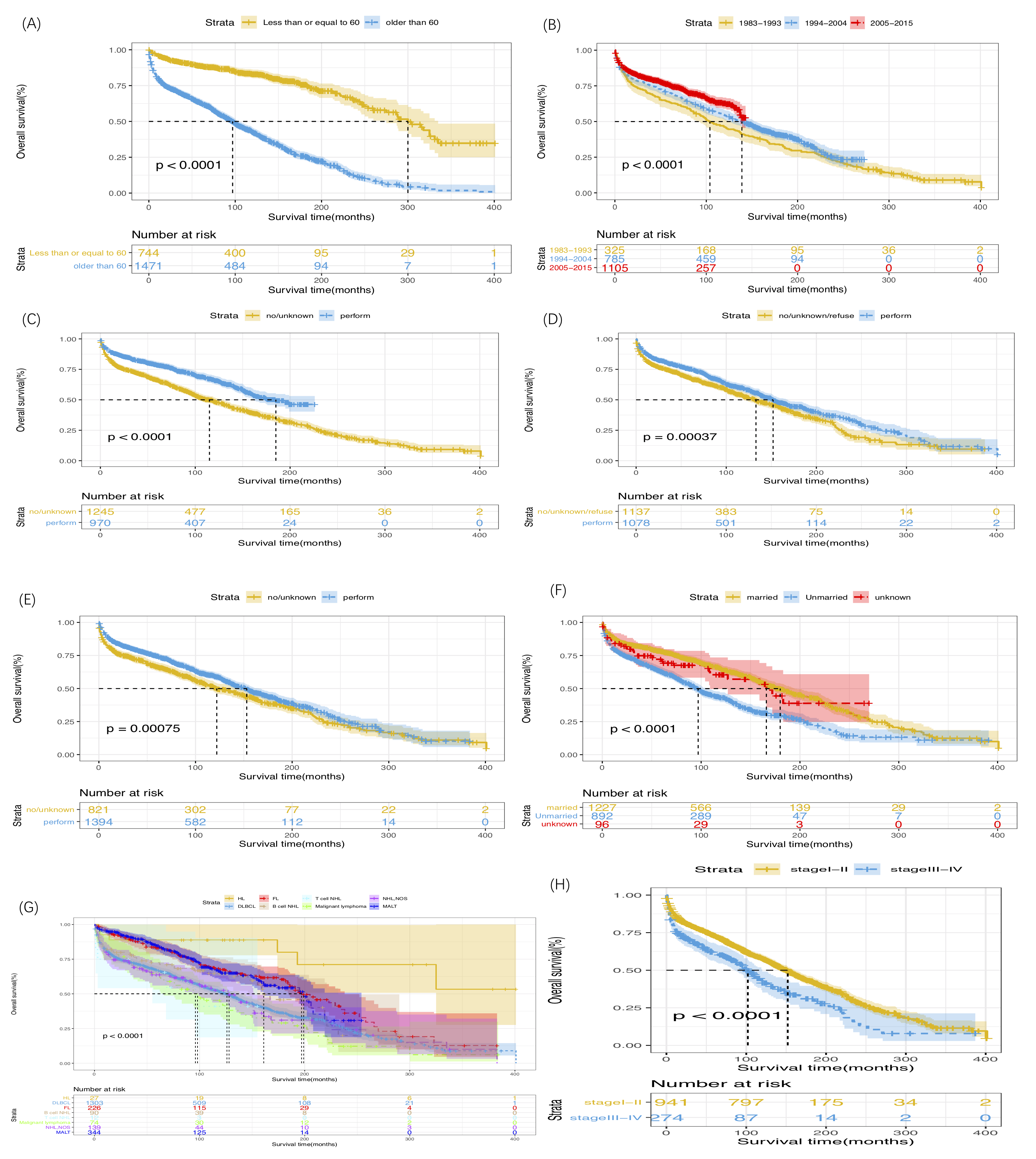

Supplement: Supplementary file 4 — Supplemental file figure 4 | Kaplan–Meier survival analysis of overall survival according to (A) age, (B) year of diagnosis, (C) surgery, (D) radiation, (E) chemotherapy, (F) marital, (G) histology, (H) stage [file 40618_2021_1712_MOESM4_ESM.tif]

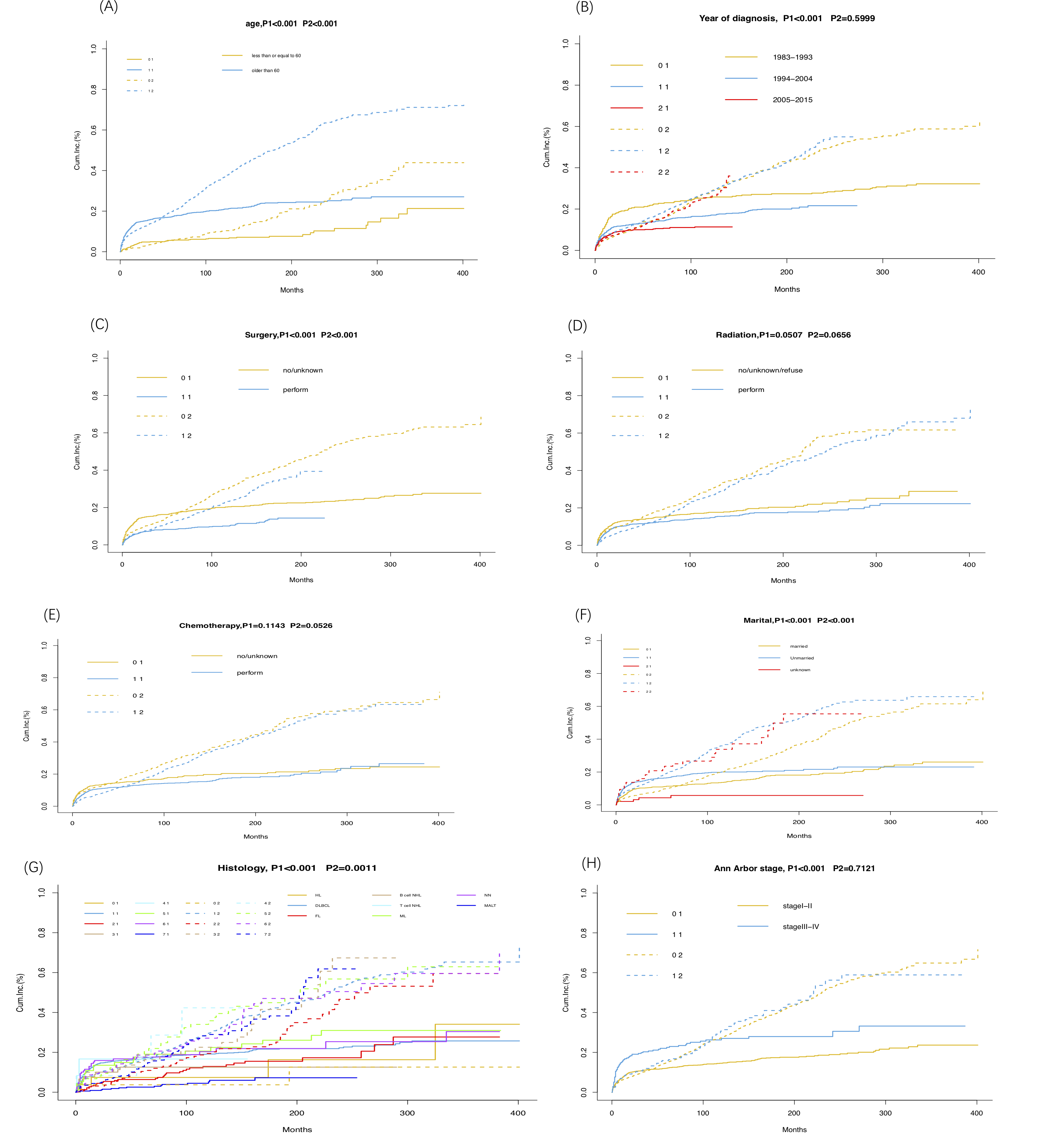

Supplement: Supplementary file 5 — Supplemental file figure 5 | Comparison of the influence of different variables on lymphoma-specific death and non-lymphoma-specific death using Gray’s test. (A) age, (B) year of diagnosis, (C) surgery, (D) radiation, (E) chemotherapy, (F) marital, (G) histology, (H) stage [file 40618_2021_1712_MOESM5_ESM.tif]
