# Supplementary material for: Prognostic evaluation models for primary thyroid lymphoma, based on the SEER database and an external validation cohort
Source: J Endocrinol Invest. 2021 Dec 4;45(4):815–24. doi: 10.1007/s40618-021-01712-3 (PMC8918170; doi:10.1007/s40618-021-01712-3)
Supplement: Supplementary file 6 — Supplementary file6 (DOCX 14 kb) [file 40618_2021_1712_MOESM6_ESM.docx]

Supplemental file

Table S1. Ann Arbor stage for PTL

| Stage | Description |
| --- | --- |
| Stage I | Disease localized to the thyroid |
| Stage II | Disease localized to the thyroid and regional lymph node basins |
| Stage III | Disease involvement on both sides of the diaphragm |
| Stage IV | Disseminated disease |
